# Using Pegylated Graphene Oxide to Achieve High Performance Solid Polymer Electrolyte Based on Poly(ethylene oxide)/Polyvinyl Alcohol Blend (PEO/PVA)

**DOI:** 10.3390/polym15143063

**Published:** 2023-07-17

**Authors:** Behnam Eslami, Ismaeil Ghasemi, Masoud Esfandeh

**Affiliations:** Faculty of Processing, Department of Plastic Processing and Engineering, Iran Polymer and Petrochemical Institute, Tehran P.O. Box 14965/115, Iran; b.eslami@ippi.ac.ir (B.E.); m.esfandeh@ippi.ac.ir (M.E.)

**Keywords:** solid polymer electrolyte, ionic conductivity, nanocomposite

## Abstract

Solid polymer electrolytes (SPEs) have emerged as a promising avenue for developing flexible lithium-ion batteries. However, the low ionic conductivity of polymers remains a primary challenge that has been the subject of intensive research efforts in recent years. In this work, polyethylene oxide (PEO), polyvinyl alcohol, lithium perchlorate (LiClO_4_), and graphene functionalized with polyethylene glycol (FGO) have been used to prepare SPE/FGO electrolytes by casting solution technique. X-ray diffraction (XRD) and differential scanning calorimetry (DSC) confirmed the reduction of SPE crystals and the increase of amorphous phases. The results demonstrated that the presence of functionalized graphene had an effective role in reducing crystallinity. Furthermore, the thermal and mechanical stability of the samples were corroborated through thermogravimetric analysis (TGA) and tensile tests, respectively. Notably, the samples exhibited adequate ionic conductivity at room temperature, with the highest ionic conductivity of 5.2 × 10^−5^ S·cm^−1^ observed for 2%wt of FGO in SPE (SPE/FGO(2)).

## 1. Introduction

The growing demand for flexible and safe batteries has driven significant interest in the development of solid polymer electrolytes (SPEs) [1]. SPEs offer a range of favorable properties, including excellent stability, flexibility, non-flammability, high specific energy, no electrolyte leakage, lightweight, and low cost [2]. However, their use is limited due to their low ionic conductivity. Polyethylene oxide is a semi-crystalline polymer proposed as one of the most important candidates for SPE due to its properties and good solubility of alkaline salts [3]. Although polyethylene oxide dissolves alkaline salts well, the presence of crystals prevents ion transfer (ion transfer occurs in the amorphous phase) [4,5]. Ionic conductivity is improved by reducing the crystal and increasing the amorphous phase. Many efforts have been made to solve this problem, including copolymerization [6,7], branching [8,9], blending [10,11], and incorporating nanoparticles [12,13]. It is important to note that there is an inverse relationship between ionic conductivity and mechanical properties. Specifically, reducing crystal formation may result in a loss of mechanical properties [14]. Therefore, any methods employed to improve ionic conductivity must also consider their potential impact on mechanical properties, aiming to either increase or maintain them without compromise.

Blending two homopolymers can result in unique properties that are superior to those of the individual homopolymers. This blending process can lead to a reduction in crystallinity and an increase in ionic conductivity by enhancing the amorphous states of the resulting blend. Kesavan et al. prepared a blend of polyethylene oxide/polyvinyl pyrrolidone (PEO/PVP) with different concentrations of lithium perchlorate. The results showed that the maximum ionic conductivity of 0.2307 × 10^−5^ S·cm^−1^ for PEO (90%)/PVP (10%)/LiClO_4_ (8%) at 30 °C [15]. Zhu et al. used amorphous polypropylene carbonate to prepare a blend with PEO. According to their report, the use of amorphous polypropylene carbonate decreased the crystallinity and increased the ionic conductivity. The blend’s maximum ionic conductivity (2.04 × 10^−5^ S·cm^−1^) occurred at 25 °C when the polypropylene carbonate component reached 50% [11]. Mallaiah et al. prepared polyethylene oxide (PEO)/polyvinylidene fluoride (PVdF) blend with sodium nitrate salt by solution casting method. They reported that the ionic conductivity of the blend with temperature was consistent with Arrhenius behavior, and the maximum ionic conductivity of 9.334 × 10^−5^ S·cm^−1^ occurred for PEO/PVdf/NaNO_3_ (80:20:5) at room temperature [16]. Polyvinyl alcohol (PVA) is a semi-crystalline polymer that has received less attention in blends with polyethylene oxide. The hydroxyl pendant groups in the chain backbone are sources of hydrogen bonding, which can assist in the formation of the blend [17]. Sarada et al. prepared a blend of PEO/PVA with KIO_3_ salt by solution casting method. Their article reported that the PEO/PVA/KIO_3_ (35:35:30) blend was the most stable [18].

As previously mentioned, incorporation of nanoparticles into the bulk of solid polymer electrolytes (SPEs) is one approach to enhancing ionic conductivity. Graphene, a carbon allotrope with a two-dimensional honeycomb structure, has garnered significant attention as a potential nanomaterial for SPE construction in recent years due to its extraordinary and unique properties [19,20,21]. Due to its hydrophobic nature, graphene exhibits weak interactions with polar polymers, such as PEO, which can result in inadequate dispersion. As a result, many studies have utilized graphene oxide or modified graphene oxide due to their enhanced solubility and ability to form stable dispersions with polar polymers. Wen et al. incorporated GO nanosheets in PEO. The ionic conductivity reached 1.54 × 10^−5^ S·cm^−1^ by embedding 1 wt% GO, which was 7 times higher than the electrolyte without GO [22]. Bao et al. synthesized modified graphene oxide by The oxyethyl-containing poly(ionic liquids) (*ox*-PIL@GO). By incorporating *ox*-PIL@GO in PEO, PEO/LiTFSI/*ox*-PIL@GO (LiTFSI: Lithium bis(trifluoromethanesulfon)imide)was prepared as a solid polymer electrolyte. The maximum ionic conductivity was obtained at 1.01 × 10^−4^ S·cm^−1^ [23]. Gomari et al. prepared SPE by grafting polyethylene glycol (PEG) to graphene oxide (FGnP) and incorporating it into PEO. Investigations showed that with 0.5% FGnP, the ionic conductivity was improved by 1 order magnitude [24].

In this study, graphene oxide was functionalized via an amidation reaction with polyethylene glycol (PEG). The resulting functionalized graphene oxide (FGO) was then dispersed at various concentrations within the PEO/PVA/LiClO_4_ electrolyte system using a solution casting method. The impact of FGO on the crystal structure, thermal properties, ionic conductivity, and mechanical properties of the SPEs was thoroughly investigated. Finally, the relationships between these properties were evaluated to gain a comprehensive understanding of the impact of FGO on SPE performance.

## 2. Experimental

### 2.1. Material

PEO (MW = 100,000 g/mol) and PVA (MW = 89,000–98,000 g/mol) prepared from Sigma-Aldrich (USA) company were used as raw materials and bases for blend preparing. LiClO4-grade battery material was purchased from Sigma-Aldrich company and was dried to vacuum for 48 h at 40 °C. The required graphite (G) was purchased from XG Science Company, USA, and was used to prepare graphene oxide through the Hammers method. Acetonitrile (98%), sulfuric acid (H_2_SO_4_, 98%), sodium nitrate (NaNO_3_), hydrogen peroxide (H_2_O_2_), and potassium permanganate (KMnO_4_) were obtained from Merck company. PEG (MW = 400 g/mol) and dehydrating agents of N, N-dicyclohexylcarbodiimide (DCC), were prepared by Sigma-Aldrich (USA) company to functionalize graphene oxide.

### 2.2. Preparation of Graphene Oxide (GO)

The Hummers method was used to prepare graphene oxide from graphite. In this method, 1 g of graphite (G) with 0.5 g of NaNO_3_ and 23 mL of 96% sulfuric acid were mixed and placed in an ice bath. Then 3 g of KMnO_4_ was added and stirred to reach ambient temperature. The suspension was then diluted and washed with 30 mL of H_2_O_2_, and the pH was neutralized. The resulting product was then centrifuged and dried in a vacuum oven at 60 °C to obtain graphite oxide powder. Graphene oxide was obtained using sonication.

### 2.3. Preparation of Functionalized Graphene (FGO)

Polyethylene glycol (PEG) has been successfully grafted onto graphene oxide (GO) nanosheets through Fischer esterification, using dicyclohexylcarbodiimide (DCC) as the leaving group in the carboxylic acid. The esterification reaction was carried out under low-temperature conditions. A mixture of 0.5 g of GO and 40 mL of dried tetrahydrofuran (THF) was added to a flask, followed by the addition of 2 g of PEG400 and 0.5 g of DCC. The mixture was then sonicated for 15 min, and subsequently refluxed under nitrogen gas for 48 h at high stirring speed. After completion of the reaction, the resulting product was separated by filtration through a 0.45 μm PTFE filter and washed with ethanol and distilled water. To ensure the complete removal of any excess or unreacted PEG, the resulting solid was centrifuged five times. Finally, the obtained precipitate was dried under vacuum at 60 °C for 24 h [24].

### 2.4. Preparation of Polymer Electrolytes

PEO and PVA, 1 g of each, were dissolved separately at 50/50 weight in 50 mL of distilled water. The polymer mixing was then performed by mixing both solutions using a magnetic stirrer. This process was continued at ambient temperature for 24 h to obtain a homogeneous and viscous solution. LiClO_4_ salt was added to the system at 8% by weight and stirred at room temperature for 24 h to obtain a polymer electrolyte. After preparation, the polymer solution was poured into a Petri dish and allowed to dry at room temperature to form films. These films were then transferred to a desiccator to continue drying. To prepare nanocomposites, the SPE was modified by incorporating FGO at varying weight percentages of 0.5%, 1%, 2%, and 3%, respectively designated as FGO(0.5), FGO(1), FGO(2), and FGO(3).

### 2.5. Characterization

Fourier transform infrared spectroscopy (FT-IR, Bruker EQUINOX 55) was carried out in the wavenumber range 4000–400 cm^−1^ with a resolution of 4 cm^−1^. The graphene samples were incorporated in dried KBr pellets, while the thin layers of polymer electrolytes were coated on KBr pellets.

X-ray diffraction (XRD) was conducted on Siemens D5000, equipped with a Cu Kα radiation source (*λ* = 0.154 nm). The d-spacing between the diffracting planes obtained by Bragg’s law (Equation (1)), and the degree of crystallinity (χ_c_) was calculated by Equation (2):

(1) $$\mathrm{d-}\mathrm{spacing}=\frac{\lambda }{2sin\left(\theta \right)}$$

(2) $$\chi_{c}=\frac{area\;of\;the\;crystalline\;peaks}{total\;area\;of\;crystalline\;and\;amorphous\;peaks}\times 100$$

Thermogravimetric analysis (TGA) was performed on the graphene samples and nanocomposites by a thermogravimetric analyzer (TGA/DSC1, Mettler Toledo, Switzerland) in the temperature range of 25–600 °C at the heating rate of 10 °C/min in a nitrogen atmosphere.

The differential scanning calorimetry (DSC) scan was used by a calorimeter (Mettler Toledo, Switzerland) for nanocomposites to evaluate thermal behavior at a heating rate of 10 °C/min in a nitrogen atmosphere. The reference material used in this test was quartz.

The universal testing instrument (STM-20, SANTAM, Iran) was used to measure the mechanical properties of SPEs, such as tensile strength and elongation at break under ASTM D882 with a strain rate of 40 mm·min^−1^. The size of the samples was 5 mm × 25 mm × 0.2 mm, and reliable data were obtained by an average of three measurement replicates.

Four-point probe method (BK Precision, USA) was conducted to measure the electrical conductivities of Blend/FGO nanocomposites. The reported data were the average of three measurements at different points.

The ionic conductivity of SPEs was conducted on an AUTO-LAB PGSTAT potentiostat/galvanostat analyzer. The measurement frequency range was between 1 MHz and 10 Hz, and the AC amplitude was 20 mV. The SPEs film is placed between two stainless steel blocking electrodes, and the ion conductivity (σ) is acquired from the Nyquist plot according to Equation (3):

(3) $$\sigma =\frac{d}{A\;\times \;R}$$

where d, A, and R are the SPE film’s thickness, the electrodes’ area, and the electrolyte resistance obtained from impedance measurement, respectively. All samples were measured at room temperature.

## 3. Results and Discussion

**Figure 1:**
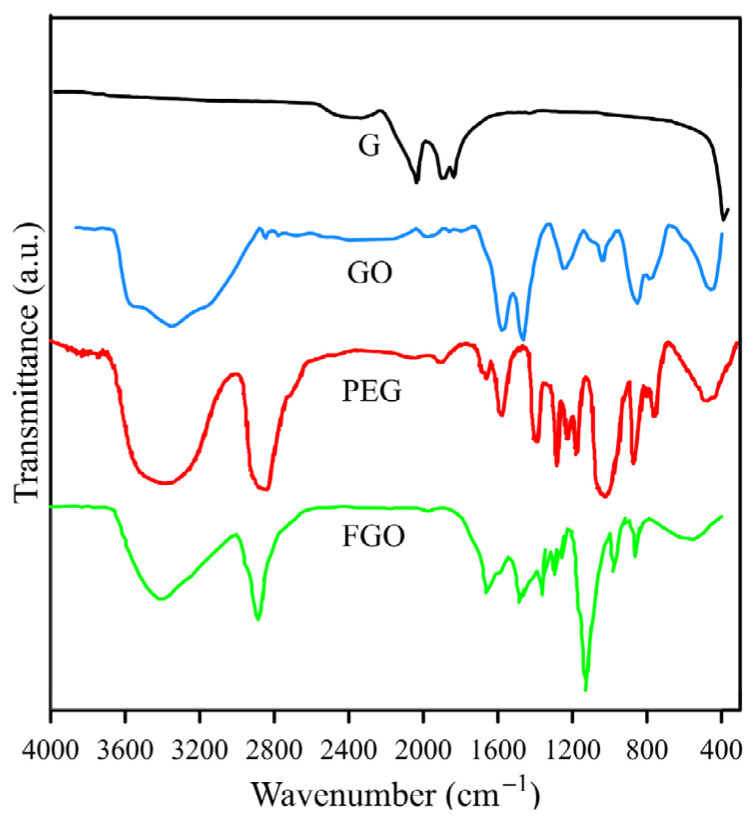
FT-IR spectra of G, GO, PEG, and FGO.

FT-IR spectra of G, GO, PEG, and FGO are presented in Figure 1. No characteristic peaks exist in the G spectrum, while significant changes were observed for the GO spectrum after its oxidation. For the GO spectrum, the broad peak at 3356 cm^−1^ is related to the stretching vibration mode of hydroxyl groups and the water absorption by KBr pellets. The strong bands at 1601 and 1500 cm^−1^ are attributed to C=O and C=C stretching vibrations, respectively. The bands at 1232, and 1034 cm^−1^ are ascribed to stretching vibration modes of epoxy (C–O–C) and alkoxy groups (C–O) [25,26]. In order to evaluate the success of the chemical modification of the GO surface with a PEG modifier, it can be useful to characterize the PEG spectrum. In the PEG spectrum, the stretching vibration of hydroxyl groups at 3350 and the asymmetric and symmetric stretching vibration of methylene groups at 2800–3000 are observed. Another characteristic peak of PEG is a broad band with strong intensity at 1034 cm^−1^, representing the stretching vibration of the ether group (C-O-C) [27]. Following the chemical modification of graphene oxide (GO) with the PEG modifier, the FGO spectrum exhibited significant changes relative to the GO spectrum. Three primary changes were observed in the FGO spectrum: (1) The appearance of band in the 3000–2800 cm^−1^ range, which is related to the methylene groups of the modifier chain; (2) A blue shift of the carbonyl stretching vibrations from 1601 cm^−1^ in GO to 1663 cm^−1^ in FGO, attributed to the formation of an ester group via the reaction of the hydroxyl groups of PEG with the carboxylic acid on the graphene oxide surface; (3) The observation of an ether band of PEG at 1128 cm^−1^ in the FGO spectrum. Based on these three changes, the chemical modification of GO with PEG can be deemed successful [24,28].

**Figure 2:**
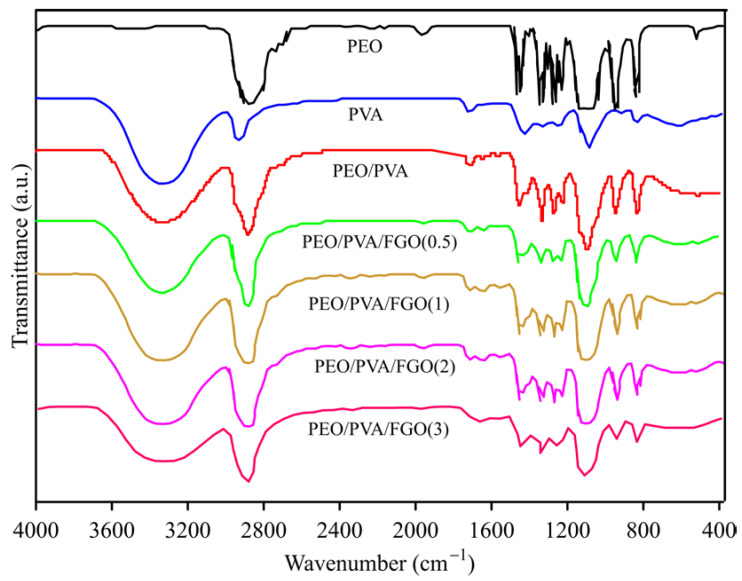
FT-IR spectra of PEO, PVA, PEO/PVA, PEO/PVA/FGO(0.5), PEO/PVA/FGO(1), PEO/PVA/FGO(2), PEO/PVA/FGO(3).

The presence of PEG on the surface of graphene leads to an improvement in the interaction between the nanolayers and the blend. On the other hand, oxygen-containing groups on the surface of graphene cause hydrogen bonding with the matrix. FT-IR analysis can be a powerful tool for investigating the interaction between FGO nanolayers with the PVA/PEO blend. FT-IR spectra of PEO, PVA, PEO/PVA, PEO/PVA/FGO(0.5), PEO/PVA/FGO(1), PEO/PVA/FGO(2), PEO/PVA/FGO(3) are shown in Figure 2. The characteristic bands of the PEO spectrum appeared at 2866, 1463, 1345, 1280, 1230, 1103, 953, and 838 cm^−1^. The broad peak at 2866 cm^−1^ is related to the asymmetric stretching vibration of C–H methylene groups. The observed peaks at 1436 and 1345 cm^−1^ can be assigned to the bending vibration of the amorphous phase, and 1280 and 1230 cm^−1^ are assigned to twisting modes of methylene groups. Two bands at 950 and 844 cm^−1^ can be related to rocking modes of CH_2_, which pertained to the helical structure of PEO polymer [29,30]. For the PVA spectrum, the broad peak at 3335 cm^−1^ represents the stretching vibration of hydroxyl groups. The absorption peaks at 2927 and 1082 cm^−1^ connect with stretching vibrations of CH_2_ and CO, respectively. The band at 1723 cm^−1^ is due to the carbonyl stretching vibrations of acetate, which remain ins PVA [31]. In the PEO/PVA spectrum, the position of the carbonyl peak is red-shifted to 1717 cm^−1^. On the other hand, the intensity of the ether peak has increased at 1088 cm^−1^, and the methylene stretching vibrations of PEO and PVA are observed at 2871 and 2949 cm^−1^, respectively, which is accompanied by a blue shift compared to their pure samples. The mentioned bands indicate an excellent mixing of PEO and PVA [30]. In the nanocomposite spectra, as the content of FGO nanolayers in the blend increases, the intensity of the FGO carbonyl peak at 1635 cm^−1^ increases. The red shift of the carbonyl wavenumber in the mixture (1635 cm^−1^) compared to the pure FGO in Figure 1 (1663 cm^−1^) and also the blue shift of their hydroxyl band (3346 cm^−1^) compared to the PEO/PVA (3330 cm^−1^) indicates a strong hydrogen bond interaction between PEO/PVA and FGO [32,33].

**Figure 3:**
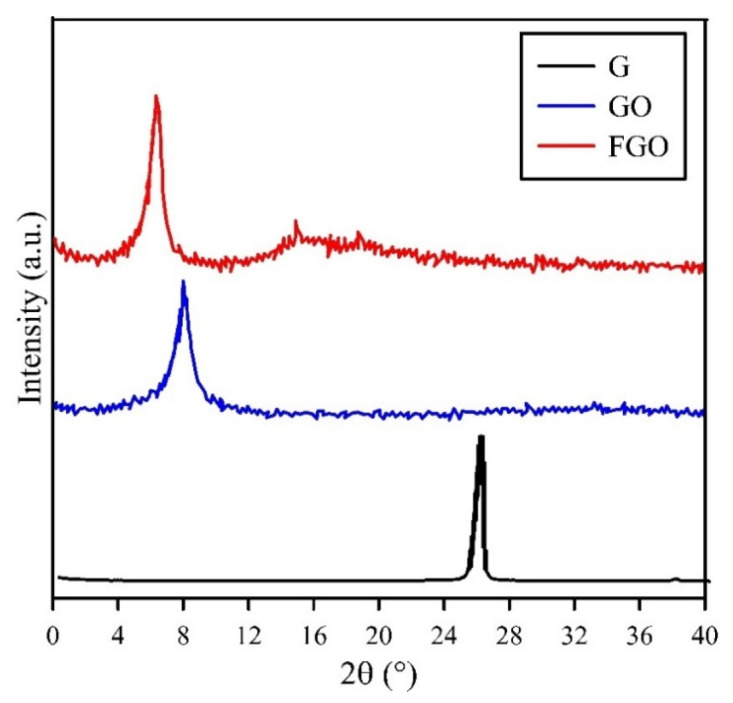
XRD patterns of G, GO, and FGO.

The XRD patterns of G, GO, and FGO are demonstrated in Figure 3. For G patterns, the single peak at 2θ = 25.9° is related to (002) basal plane (d-spacing = 0.34 nm) [34]. For the GO pattern, due to the presence of oxygen-containing groups on the graphene surface, the desired peak shifted to 2θ = 7.6° (d-spacing = 1.1 nm), which indicates the exfoliation of nanolayers [35]. For the FGO pattern, by grafting PEG on the graphene surface, the peak shifted to 2θ = 6°. The diffraction angle decreased, indicating an increase in the d-spacing between the nanolayers compared to GO. A weak and broad peak at 2θ = 16.6° is due to the re-clustering of nanolayers [36].

**Figure 4:**
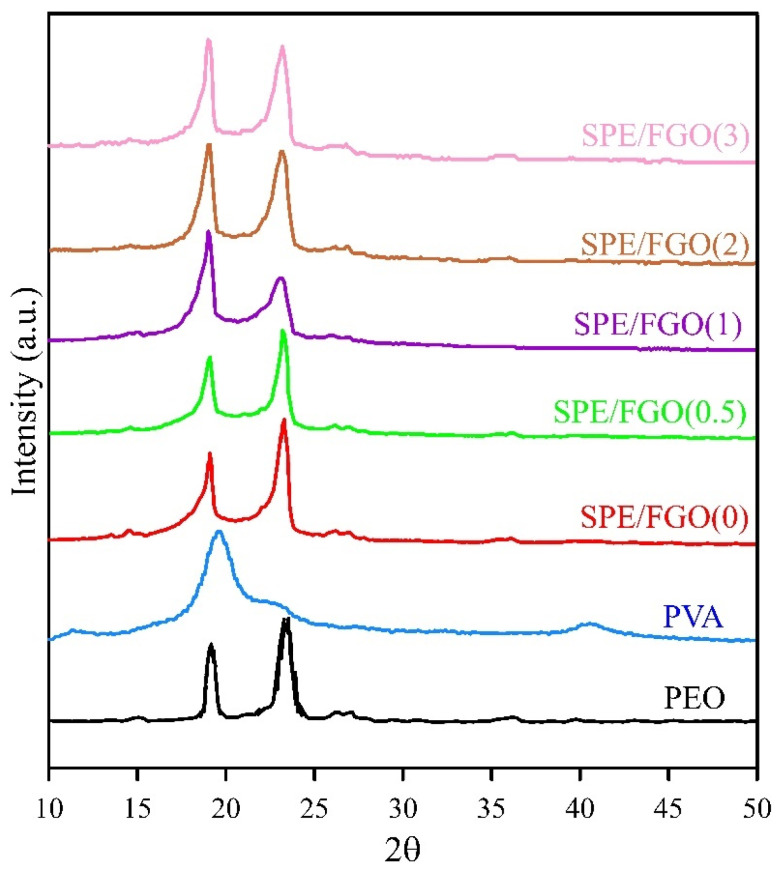
XRD patterns of pure PEO, pure PVA, SPE/FGO(0), SPE/FGO(0.5), SPE/FGO(1), SPE/FGO(2), SPE/FGO(3).

Figure 4 represents the XRD patterns of PEO, PVA, and SPEs. For the PEO pattern, the characteristic peaks at 2θ = 19 and 23.5° are corresponded to (120) and (112) reflection planes, respectively. These peaks show the semi-crystalline nature of PEO. PVA’s semi-crystalline nature has led to a broad peak at 19.5 and 22.6°, which corresponds to (101) and (10$\bar{1}$) planes, respectively. For the SPE/FGO(0) pattern, by merging two pure spectra, two peaks at 2θ = 19.1 and 23.4° correspond to the effect of two polymers with each other [37]. The degree of crystallinity of PEO, PVA, and SPEs are listed in Table 1. According to Table 1, embedding FGO nanolayers in SPE reduced the degree of crystallinity of the nanocomposite. In all SPEs, the characteristic peak of FGO is not observed, which indicates the excellent dispersion of FGO in the SPE bulk [38].

**Figure 5:**
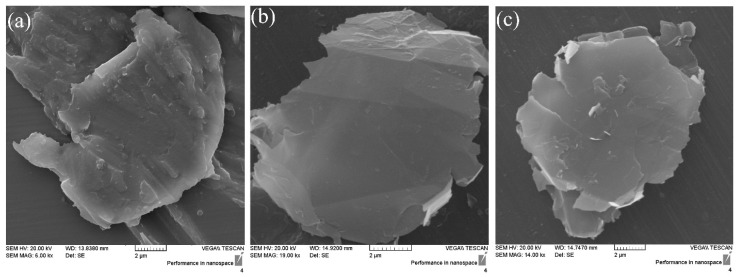
The SEM images of (**a**) G, (**b**) GO and (**c**) FGO.

The SEM images of G, GO and FGO are shown in the Figure 5. The G nanolayers have accumulated and formed clusters due to the absence of functional groups. For GO, the oxygen groups on the graphene surface caused the graphene sheets to be completely separated from each other and become a single sheet. Also, the sharp edges of GO indicated the presence of oxygen groups on the surface. But in FGO, functionalized graphene sheets are slightly rounded, which is due to the chemical reduction by pegylation. By comparing the size of the sheets in all three cases, it can be seen that the oxidation and functionalization of nanoparticles do not have much effect on the size of the graphene sheets.

**Figure 6:**
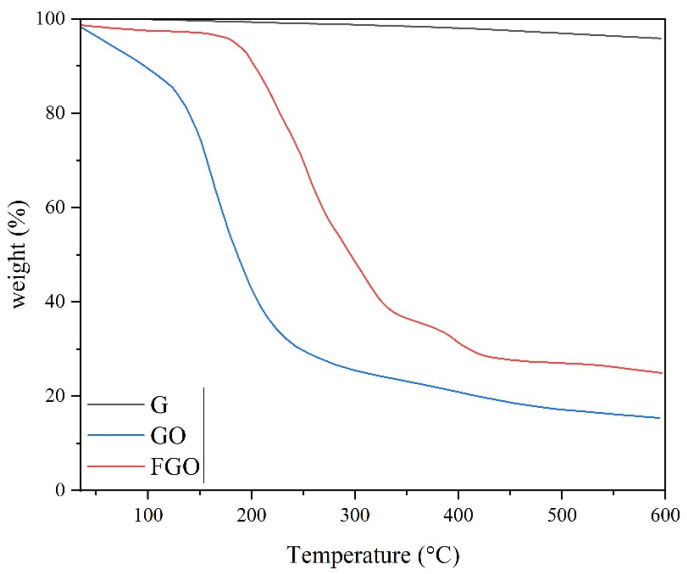
TG curves of G, GO, and FGO.

To evaluate the thermal stability of graphene types and nanocomposites, TGA was used. Figure 6 illustrates the TG curves of G, GO, and FGO. For G, no weight loss was observed until the temperature of 600 °C. A significant drop in the onset temperature of 150 °C has been observed for the GO. The weight loss below 150 °C is related to the reduction of water remaining between the GO layers, and weight loss above 150 °C is related to the decomposition of oxygen-containing groups on the surface of GO [39]. The successful oxidation of graphene nanolayers can be proved by comparing the TG curves of G and GO. The extreme weight loss of the GO to the char residual of 15.33% indicates the inappropriate thermal stability of GO nanolayers, but the FGO had higher stability than GO [40]. For FGO, the onset decomposition temperature was shifted to 200 °C. The increase of 50 °C in the onset decomposition temperature of FGO compared to GO indicates that the thermal stability of nanolayers with PEG grafting on GO nanosheets was improved. The TG diagram of the FGO sample shows two degradation steps in the temperature ranges of 220–330 °C and 350–450 °C. The first step, which has the largest decrease, is related to the degradation of oxygen-containing groups on the surface of graphene. The second step is related to the degradation of PEG [41,42].

**Figure 7:**
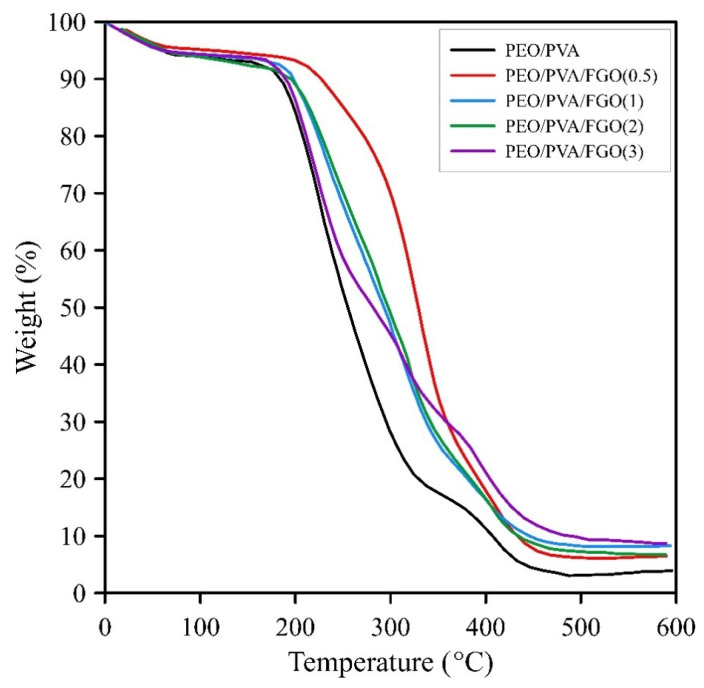
TG curves of PEO/PVA, PEO/PVA/FGO(0.5), PEO/PVA/FGO(1), PEO/PVA/FGO(2), PEO/PVA/FGO(3).

The thermal stability of PEO/PVA blend and nanocomposites was investigated by TGA. TG plots are shown in Figure 7. The temperature of 5% weight loss (T_5%_), 25% weight loss (T_25%_), and the char residual are listed in Table 2. Two tangent lines have been drawn on the curve in the region where the weight loss occurs to calculate T_5%_, known as the onset degradation temperature. The point where these two tangent lines intersect corresponds to the T_5%_ temperature, which means the temperature at which 5% weight loss has occurred. It is a common method in the literature to report the onset degradation temperature [43]. Results have shown a 4.3% downturn due to all specimens’ evaporation of the remaining moisture. Two degradation steps were observed for the PEO/PVA sample. The first step of degradation that occurred at 188–328 °C was related to the decomposition of the PVA chain, and the second step in the range of 343–444 °C was related to the decomposition of PVA and PEO chains [44,45]. Higher thermal stability was observed for nanocomposites compared to PEO/PVA by increasing the FGO content. According to Table 1, 0.5% FGO content showed the highest values of T_5%_ and T_25%_, which increased by 23.6 and 74.8 °C, respectively, compared to PEO/PVA. T_5%_ and T_25%_ nanocomposites containing 1–3% FGO were lower than 0.5% FGO, which is attributed to the aggregation of FGO and the decrement of the interaction between the nanolayers and the matrix. Char residual is proportional to the FGO contents. The increase in FGO amounts led to a char residual increase. Graphene nanolayers prevent the release of volatile gases due to their planar structure, which can be described as follows: (1) Appropriated interaction between the matrix and the nanolayers causes the restriction of polymer chain mobility. The low mobility of the chains prevents the release of volatile gases; (2) The presence of sheets in the polymer matrix causes the release of volatile gases in curvy paths, which causes a delay in thermal degradation [46].

On the other hand, the chemical structure of graphene nanolayers has a radical-scavenging nature. The trapping of radicals causes a delay in thermal degradation [47]. The good thermal stability of nanocomposites shows that a proper interaction between the matrix and FGO has been established by grafting PEG on the surface of graphene. The thermal properties of nanocomposites showed that they could be a suitable alternative to liquid electrolytes of lithium-ion batteries.

**Figure 8:**
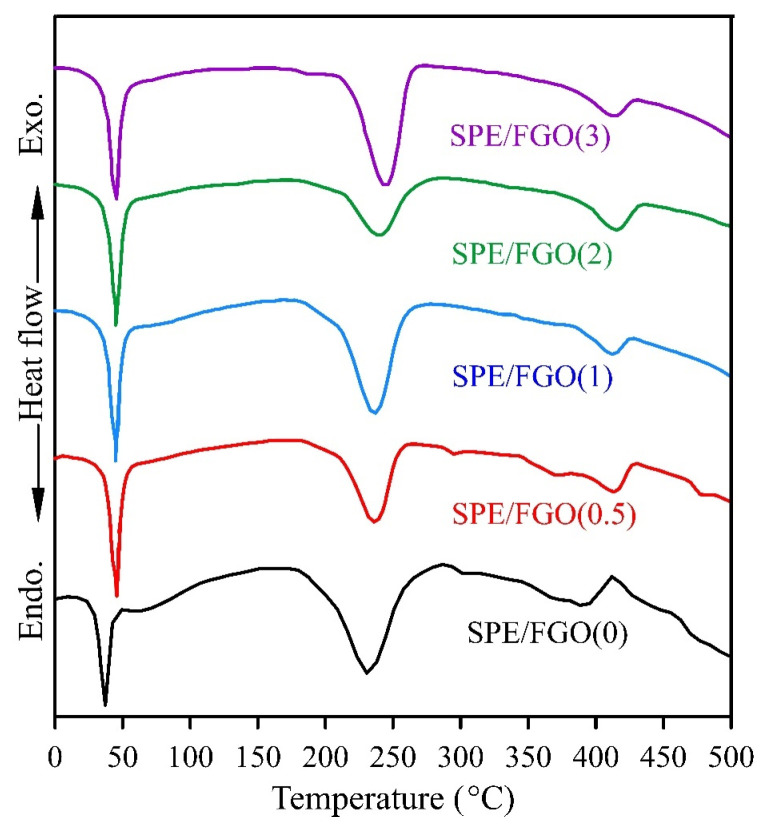
DSC thermograms of SPEs with Different Weight Percentages of FGO.

The DSC analysis is a practical and helpful tool for investigating the thermal properties of polymers. The DSC curves of SPEs are demonstrated in Figure 8, and the acquired data are illustrated in Table 3. According to Table 3, the melting temperature (T_m_) of specimens has not changed and is almost constant. Concomitantly, the degradation endothermic peak (T_d_) exhibited a significant increase with the rise in FGO concentration. Notably, the highest T_d_ value was observed for the SPE/FGO(0.5) sample, indicating superior thermal stability relative to the blend. These findings were further supported by the results obtained from TGA. Abd El-kader et al. observed that the melting temperature of PEO overlapped with the glass transition temperature (T_g_) of PVA in the DSC curves of the PEO/PVA blend [48].

For this reason, they introduced the endothermic peak at 65 °C as T_g_ of the blend [48]. According to Table 3, Tg increased with the increase of FGO contents in the blend. The increase in Tg was due to the proper interaction of the matrix and graphene nanolayers, which caused a decrease in chain mobility and led to an improvement in thermal stability [49]. To investigate the effect of graphene nanolayers on the crystalline properties of the blend, the degree of crystallinity was determined from the following equation [24]:

(4) $$\chi_{c}=\frac{\Delta H_{m}}{\Delta H_{m}^{{}^{\circ}}}\times 100$$

where χ_c_, ΔH_m_, ΔH°_m_ are the degree of crystallinity, melting enthalpy of PVA, and melting enthalpy of PVA at 100% crystallinity of pure PVA (ΔH°_m_ = 142 j/g) [50], respectively. As mentioned, since the T_m_ of PEO overlaps with the Tg of PVA polymer, it will be challenging to accurately determine the melting enthalpy of PEO; therefore, studying the degree of crystallinity of PVA will be more accurate and efficient. The decrease in the degree of crystallinity of the SPEs compared to the SPE/FGO(0) indicates that the appropriate dispersion of graphene nanolayers prevents crystal formation [24]. The degree of crystallinity is in good agreement with XRD analysis, and the results of DSC, like XRD, show a decrease in the crystallinity of nanocomposites.

To investigate the applicability of graphene-containing nanocomposites as polymer electrolytes, it is necessary to measure the graphene concentration at which the electrical percolation threshold occurs. The SPEs must be electrically insulating to prevent a short circuit in the battery. Studying their electrical properties helps to determine this behavior. The electrical conductivities of nanocomposites were studied and are listed in Table 4. As can be seen, all the samples had low electrical conductivity, and none showed the electrical threshold. It can be pointed out that, in order to reach the electrical threshold, two suitable good dispersion and plate-to-plate contacts must be established [51]. However, there was no noticeable change in the electrical conductivity of the nanocomposites despite the appropriate exfoliation and dispersion of FGO, which is due to the oxidation and functionalization of graphene, which caused defects in the chemical structure of graphene and greatly reduced the electrical properties of graphene [52].

**Figure 9:**
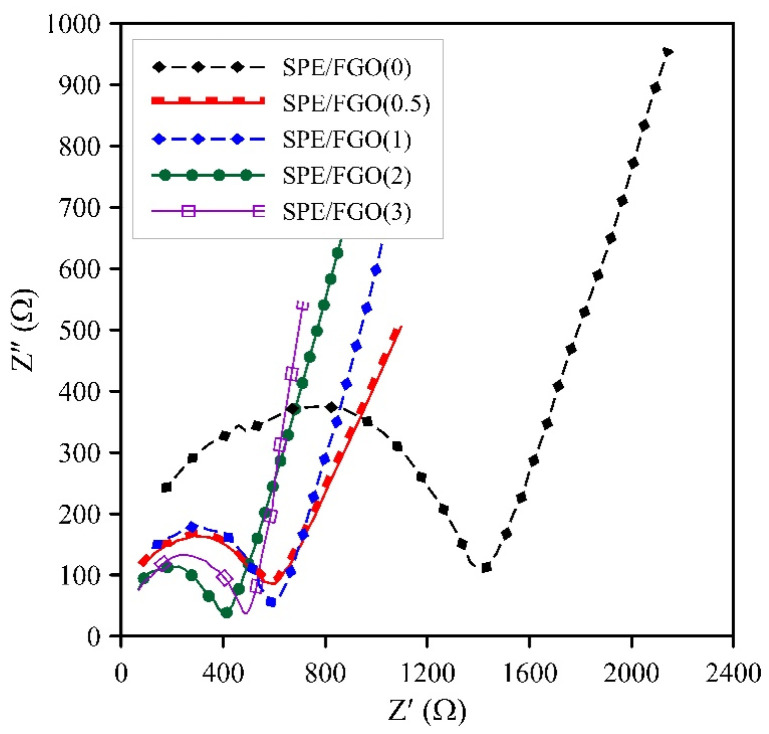
Nyquist plots of SPEs with Different Weight Percentages of FGO at room temperature.

The ionic conductivity was utilized by impedance spectroscopy (EIS) to evaluate the efficiency of solid polymer electrolytes, and the effect of adding graphene nanosheets was studied. Nyquist plot of SPEs is illustrated in Figure 9. The bulk resistance (Rb) is obtained from the intersection of the fitted semicircle with the *Z*’ axis [16]. The ion conductivity was calculated according to Equation (3), considering each sample’s thickness is 200–300 μm. The results are collected in Table 4.

As can be seen, the lowest bulk resistance is related to the SPE/FGO(2) sample, so the highest ionic conductivity belongs to this sample. Gomari et al. reported the ionic conductivity of PEO was 1.31 × 10^−6^ S·cm^−1^ [24]. According to Table 4, blending PEO with PVA (50:50, *w*/*w*) played an influential role in ionic conductivity, and its ionic conductivity increased to 1.2 × 10^−5^ S·cm^−1^. The one-order increase in ionic conductivity implies that the blending impacts the reduction of crystals and the increase of amorphous regions, which was previously confirmed by XRD and DSC results. Regarding Table 4, the ionic conductivity has increased upon increasing FGO concentration to 2%, but it has decreased in the SPE/FGO(3). The proper dispersion of nanosheets and access to a larger specific surface area in the matrix caused the PEG chains to have a strong interaction with the blend through hydrogen bonding, which led to a reduction in the growth of spherulites. For the SPE/FGO(3), crystal growth was provided by aggregating nanolayers and decreased ionic conductivity. The noteworthy point is that all SPE samples containing graphene had very high ionic conductivity compared to SPE without graphene. Several reports have related to reducing the crystallinity of solid polymer electrolytes by adding graphene and improving ionic conductivity, although the reported results had lower values than the current reports. Kuduru et al. evaluated the effect of graphene oxide on the performance of PEO/PVP/NaIO4 polymer electrolytes. They showed that the lowest crystallinity was obtained at 0.6 wt%, and the ionic conductivity at room temperature reached between 1.56 × 10^−7^ S·cm^−1^ and 1.89 × 10^−6^ S·cm^−1^ [53]. Hu et al. prepared a solid polymer electrolyte by chemically modifying graphene oxide with ionic liquid and incorporating it into PEO. The highest ionic conductivity at 1% was reported as 1.8 × 10^−5^ S·cm^−1^ at room temperature [54].

One of the most important properties of polymer electrolytic films is their mechanical stability. For this purpose, the mechanical properties of nanocomposites were investigated by tensile test. The stress–strain properties of PEO, PVA, PEO/PVA, and PEO/PVA/FGO nanocomposites such as Young’s modulus, yield stress, mechanical strength, and strain at the break are collected in Table 5. Regarding Table 5, the mechanical properties of the PEO/PVA film showed relatively intermediate properties compared to pure PEO and PVA. Young’s modulus was calculated by determining the slope of the stress–strain curves at low strains. Young’s modulus was assigned by calculating the slope of the stress–strain curves at small strains. Young’s modulus increased with increasing graphene concentration up to 1%, and a decreasing trend was observed at concentrations higher than 1%. Other determined parameters had similar behavior. The highest Young’s modulus showed an increase of 35% for the PEO/PVA/FGO(1). According to the obtained data, the tensile strength of the blend increased from 10.8 MPa (PEO/PVA) to 13.6 MPa (PEO/PVA/FGO(1)), but with higher than 1%, these properties started to decrease. Also, the strain at break of PEO/PVA/FGO nanocomposites represented an increasing trend by increasing the FGO concentration up to 1% and reaching 483%; however, the increase in the FGO concentration has stopped and has reached 386%.

Based on the previous studies conducted by other researchers, the intrinsic properties of the filler, its specific surface, and the ability to transfer load from the matrix to the nanoparticles play very important roles in determining the efficiency and mechanical properties of polymer nanocomposites. Therefore, the mechanical properties of nanocomposites enhance due to the presence of nanoparticles under two conditions: (1) excellent dispersion of nanoparticles provided in the matrix; (2) Strong interfacial adhesion established between nanoparticles and polymer chains. Because the proper dispersion and exfoliations into low-layer or even single-layer nanosheets ensure the use of the large specific surface area of these nanoparticles, strong interfacial interactions to cause optimal load transfer from the matrix to graphene nanosheets [24]. As mentioned above, the effect of nanoparticle modification on the enhancement of mechanical properties, especially tensile strength, can be justified. The terminal hydroxyl groups in PEG, which are grafted on the graphene surface, can establish strong hydrogen bonds with the oxygen atom in the ether groups of the PEO and hydroxyl group of PVA chains. Also, the second reason can be related to the strong adhesion between FGO and PEO/PVA matrix. It should be mentioned that another factor that affects the mechanical properties of semi-crystalline polymers is the degree of crystallinity. Indeed, the higher degree of crystallinity increases the stiffness and strength due to the reinforcing effect of the crystals. The DSC revealed that adding FGO to the PEO/PVA blend reduces crystallinity. As a result, other factors, including the high surface-to-weight ratio of graphene sheets and its excellent inherent mechanical properties, along with appropriate dispersion and adhesion, have played a more influential role in improving the mechanical performance of PEO/PVA nanocomposites.

Table 6 presents a comparison of the ionic conductivity of solid polymer electrolytes in various articles with the current research. Based on the comparison with previous studies, it has been demonstrated that the ionic conductivity in the current research has been more effective due to the presence of graphene and PVA in PEO. This implies that incorporating graphene and PVA into PEO has led to an increase in the ionic conductivity of solid polymer electrolytes.

## 4. Conclusions

In this study, the impact of PEG-grafted graphene on the thermal, mechanical, and electrical properties of a PEO/PVA blend, with lithium perchlorate salt as a solid polymer electrolyte, was evaluated. FT-IR, XRD, and TGA were performed to assess the success of the chemical modification of the nanoplatelets. The results of these analyses confirmed the successful chemical modification of the graphene nanoplatelets. The FT-IR analysis of the PEO/PVA/FGO nanocomposite discussed the effect of hydrogen bonding through the shifts in the wavenumbers of the hydroxyl and carbonyl peaks. XRD and DSC confirmed the presence of FGO in the SPE, which reduced crystallinity. Additionally, TGA demonstrated that the thermal stability of the SPEs increased with the presence of graphene. The insulating properties of the SPEs were evaluated and found to exhibit no electrical threshold up to 3% graphene concentration, attributed to the structural defects caused by graphene oxidation and chemical modification reactions. Ionic conductivity was determined by calculating the bulk resistance obtained from EIS, with the maximum value reported as 5.2 × 10^−5^ S·cm^−1^ for the SPE/FGO(2) at room temperature. The mechanical stability of the SPEs was assessed by examining the tensile properties of SPE films, including Young’s modulus, strength, and strain at break. The proper dispersion of FGO and the excellent adhesion between the nanosheets and matrix enabled load transfer from the matrix to FGO, thereby promoting the mechanical properties of the SPEs.

## Figures and Tables

**Table 1:** Degree of crystallinity of solid nanocomposite electrolytes as calculated from XRD analsis.

Samples	Degree of Crystallinity (%)
PEO	80.5
PVA	51.5
SPE/FGO(0)	48.2
SPE/FGO(0.5)	44.3
SPE/FGO(1)	39.5
SPE/FGO(2)	34.2
SPE/FGO(3)	34.3

**Table 2:** The results were obtained from the TG curves of the blend and its nanocomposites.

Samples	T_5%_ (°C)	T_25%_ (°C)	Char Residual (%)
PEO/PVA	182.9	214.6	4.32
PEO/PVA/FGO(0.5)	206.5	289.4	6.86
PEO/PVA/FGO(1)	188.5	234	8.82
PEO/PVA/FGO(2)	195.8	240.5	7.22
PEO/PVA/FGO(3)	187.7	218.9	9.4

**Table 3:** The Data Obtained from DSC Test for SPEs with Different Weight Percentages of FGO.

Samples	T_g_ (°C)	T_m_ (°C)	T_d_ (°C)	ΔH_m_ (J/g)	χ_c_ (%)
SPE/FGO(0)	40.1	233.2	391.2	56.11	39.52
SPE/FGO(0.5)	45.65	237.6	419.9	44.63	31.43
SPE/FGO(1)	45.7	237.4	411.1	42.77	30.12
SPE/FGO(2)	46.4	241.5	416.3	29.39	20.7
SPE/FGO(3)	46.7	243.8	415.3	42.04	29.61

**Table 4:** Ionic and electrical conductivity of SPEs with Different Weight Percentages of FGO.

Samples	σ (S·cm^−1^)	Electrical Conductivity (S·cm^−1^)
SPE/FGO(0)	1.2 × 10^−5^	7.1 × 10^−12^
SPE/FGO(0.5)	2.85 × 10^−5^	5.32 × 10^−11^
SPE/FGO(1)	3.61 × 10^−5^	6.2 × 10^−11^
SPE/FGO(2)	5.2 × 10^−5^	8.4 × 10^−11^
SPE/FGO(3)	4.2 × 10^−5^	9.1 × 10^−11^

**Table 5:** Mechanical properties of pure PEO, pure PVA, PEO/PVA blend, and its nanocomposites.

Samples	Young’s Modulus (MPa)	Yield Stress (MPa)	Tensile Strength (MPa)	Elongation at Break (%)
PEO	203	6.65	7.3	583
PVA	682	16.1	16.6	211
PEO/PVA	453	10.4	10.8	374
PEO/PVA/FGO(0.5)	573	12.9	13.4	465
PEO/PVA/FGO(1)	615	13.3	13.6	483
PEO/PVA/FGO(2)	563	12.7	13.4	456
PEO/PVA/FGO(3)	521	11.3	11.4	386

**Table 6:** Comparison of SPEs with different ionic conductivity in the previous literature and SPE/FGO(2).

SPEs	Temperature (°C)	Ionic Conductivity (S/cm)	Ref.
PEO/PVA/LiOH	25	2.18 × 10^−5^	[55]
PEO/PPC	25	2.04 × 10^−5^	[11]
PEO/PVP/LiClO_4_	30	0.2307 × 10^−5^	[37]
SPE/FGnP(0.5)	25	2.53 × 10^−5^	[24]
SPE/FGO(2)	25	5.2 × 10^−5^	This work

## Data Availability

All data are included in the article.
